# Active maintenance in working memory reinforces bindings for future retrieval from episodic long-term memory

**DOI:** 10.3758/s13421-024-01596-7

**Published:** 2024-07-05

**Authors:** Vanessa M. Loaiza, Alessandra S. Souza

**Affiliations:** 1 Department of Psychology, University of Sheffield, 219 Portobello, Sheffield, S1 4DP UK; 2 Department of Psychology, University of Essex, Colchester, UK; 3 Faculty of Psychology and Education Sciences, Center for Psychology, University of Porto, Porto, Portugal; 4 Department of Psychology, University of Zurich, Zürich, Switzerland

**Keywords:** Working memory, Long-term memory, Episodic memory, Cognitive modeling

## Abstract

Many theories assume that actively maintaining information in working memory (WM) predicts its retention in episodic long-term memory (LTM), as revealed by the beneficial effects of more WM time. In four experiments, we examined whether affording more time for intentional WM maintenance does indeed drive LTM. Sequences of four words were presented during trials of simple span (short time), slow span (long time), and complex span (long time with distraction; Experiments 1–2). Long time intervals entailed a pause of equivalent duration between the words that presented a blank screen (slow span) or an arithmetic problem to read aloud and solve (complex span). In Experiments 1–3, participants either serially recalled the words (intentional encoding) or completed a no-recall task (incidental encoding). In Experiment 4, all participants were instructed to intentionally encode the words, with the trials randomly ending in the serial-recall or no-recall task. To ensure similar processing of the words between encoding groups, participants silently decided whether each word was a living or nonliving thing via key press (i.e., an animacy judgment; Experiments 1 and 3–4) or read the words aloud and then pressed the space bar (Experiment 2). A surprise delayed memory test at the end of the experiment assessed LTM. Applying Bayesian cognitive models to disambiguate binding and item memory revealed consistent benefits of free time to binding memory that were specific to intentional encoding in WM. This suggests that time spent intentionally keeping information in WM is special for LTM because WM is a system that maintains bindings.

The classic “textbook” view of human memory entails a “transfer” of the current contents of consciousness to a more permanent and durable store (Atkinson & Shiffrin, 1968; Waugh & Norman, 1965). Although many models of memory have been developed since, this fundamental idea has been influential: Many theoretical views assume that the processes involved in keeping information temporarily active and accessible in working memory (WM) likewise impact the likelihood of retrieving that information later on from episodic long-term memory (LTM). These processes have included articulatory rehearsal (Atkinson & Shiffrin, 1968; Waugh & Norman, 1965), elaborative rehearsal (Craik & Lockhart, 1972; Craik & Tulving, 1975), refreshing (Camos & Portrat, 2015; Johnson et al., 2002), and consolidation (Cotton & Ricker, 2021), among others. Rather than detail their specific operational definitions and the differences between them, for the purposes of this work we will focus on the two common assumptions among them: (1) participants actively engage the process in the service of maintaining or acting on the information held in WM, and (2) the more frequent or longer the opportunities to engage in the process in WM, the more likely it is that the information will be retained in LTM.

These assumptions are reasonable and often justified in the literature: Regarding the first assumption of *active maintenance*, manipulations intended to vary the opportunity to engage a given process in WM often show effects on LTM (e.g., Camos & Portrat, 2015; Cotton & Ricker, 2021; Jarjat et al., 2018; Johnson et al., 2002; Rose et al., 2014), and participants’ self-reported WM maintenance strategies often correlate with subsequent retrieval from LTM (e.g., Loaiza & Lavilla, 2021; Rundus, 1971; Tan & Ward, 2008). However, these effects are not always consistently demonstrated (Bartsch et al., 2018, 2019a) or conclusively causative (Souza & Oberauer, 2018, 2020), and there is often controversy over whether a given manipulation does indeed vary the process in question given that the processes are not directly observable (Oberauer et al., 2012).

Perhaps the most reliable and directly observable evidence for the importance of ongoing processing in WM for later retrieval from LTM is that of elaborative rehearsal: Asking participants to make verifiable decisions that pertain to the deep, semantic characteristics of memoranda strongly benefit delayed tests of free recall and recognition relative to shallow decisions (Craik & Tulving, 1975; Hyde & Jenkins, 1969, 1973; Loaiza et al., 2011; Rose et al., 2014). However, this levels-of-processing effect can be observed regardless of whether participants are aware of a final memory test (Craik & Tulving, 1975; Hyde & Jenkins, 1969), suggesting that the long-term benefit of elaborative rehearsal does not depend on actively trying to keep information in mind. This is at odds with the assumption of the importance of active maintenance in WM. Indeed, most models of WM assume that the processes crucial to its functioning are intentional and controlled, including those that impact later retrieval from LTM. Thus, if actively processing information in WM is important to its long-term retention, then whatever essential process this might be, it has to be intentionally applied to keeping information active in WM. Nevertheless, the evidence for this key assumption of the unique importance of active maintenance in WM is either inconsistent and sometimes controversial (e.g., Bartsch et al., 2018; Souza & Oberauer, 2018), or, in the case of elaborative rehearsal as explained here, contradictory.

There is at least ample evidence for the second assumption of the *benefits of time*: Hartshorne and Makovski’s (2019) meta-analysis of more than 60 experiments revealed that the longer an item is held in WM, the greater the likelihood that it is later retrieved from LTM. However, it is not clear exactly what participants are doing given the aforementioned persistent drawback in this literature that WM processes are not directly observable. It may be that these benefits of time do not rely on actively keeping the information in mind, but instead reflect the mere benefits of temporal distinctiveness or spacing. Indeed, spacing effects can be observed both when participants intentionally encode the memoranda for a future test and during incidental encoding in which they are unaware and do not expect a test (Cepeda et al., 2006). Combined with the previous doubts about the importance of active maintenance in WM, these results may suggest that processing information in WM is not particularly special or important for its long-term retention. This possible conclusion would dramatically shift many theoretical understandings of the importance of WM for LTM, and thus it is important to specifically test.

## Current experiments

Here, we explicitly tested the often-taken-for-granted intuition that actively keeping information accessible in WM for as long as one can is an essential driver of the benefits of time to LTM. To this end, we used a paradigm from prior work that has demonstrated the benefits of time in WM to LTM (Loaiza & Lavilla, 2021; Souza & Oberauer, 2017). In these studies, participants viewed four successively presented words in three different types of classic WM tasks: Simple span (words are presented successively without interruption), complex span (words are interleaved with a distracting arithmetic problem to read aloud and solve), and slow span (words are interleaved with a blank screen of equivalent duration to the arithmetic problem in the complex span trials). Participants recalled the words immediately at the end of each trial (to test WM) and after a delay (to test LTM). The results have generally shown that words studied during complex span and slow span (which afford longer WM time) are better remembered after a delay than words from simple span trials (shorter WM time), thus consistent with the assumption that affording greater time to process information in WM is more likely to lead to its successful retrieval from LTM. We used this paradigm to test whether this benefit of time for LTM is exclusive to when participants actively try to keep the information in WM.

**Fig. 1:**
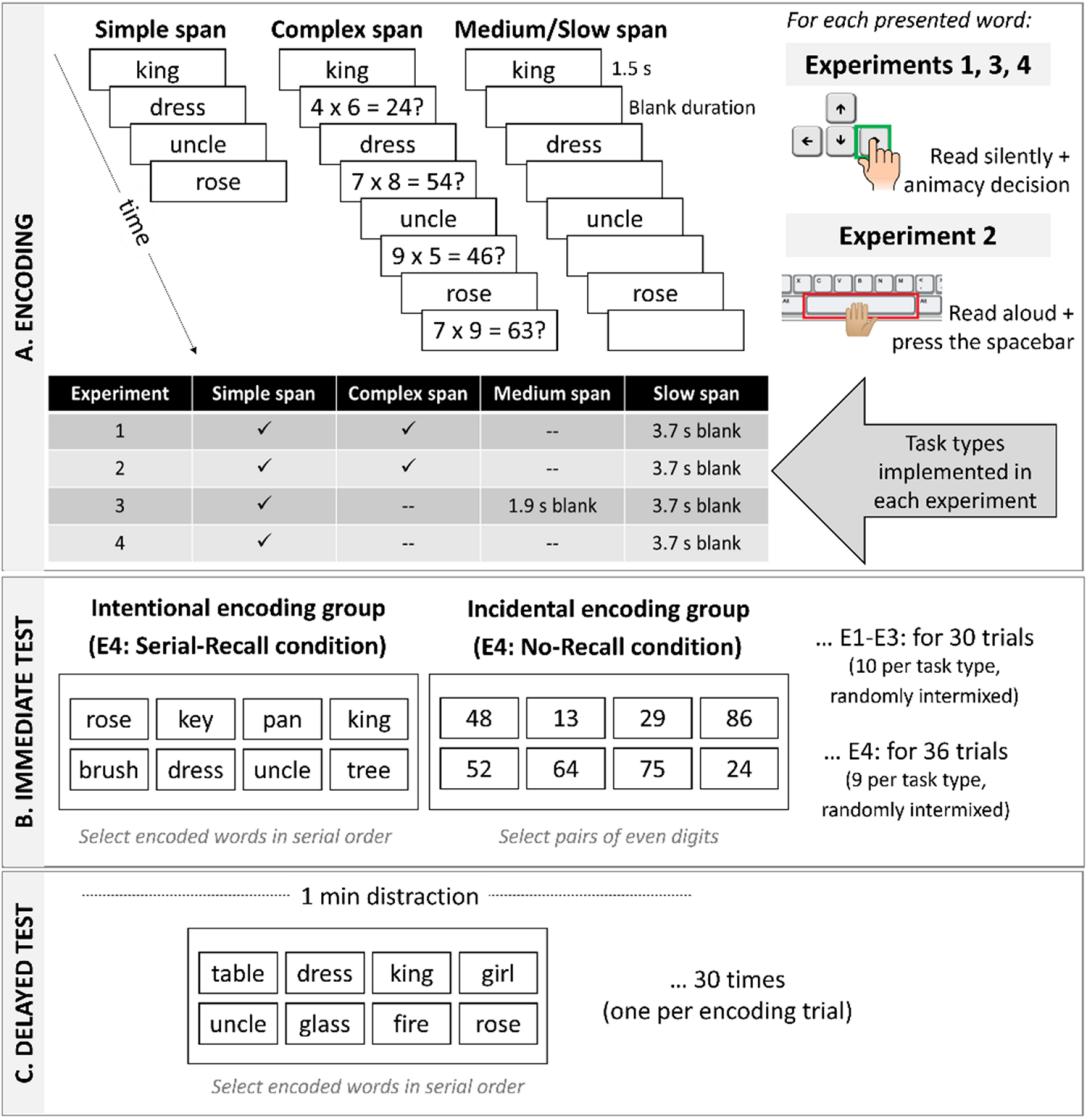
Illustration of the tasks in Experiments 1 to 4 of the encoding phase (**A**), the immediate test (**B**), and surprise delayed recall test (**C**). (Color figure online)

Figure 1 shows the tasks of Experiments 1 through 4: Participants viewed four successively presented words in trials of simple span, slow span, and complex span (Experiments 1–2). In Experiments 1–3, half of the participants were instructed to maintain these words for an immediate recall test at the end of the trial (intentional [WM] encoding group), whereas the other half of the participants completed an unrelated no-recall task at the end of each trial (incidental [no-WM] encoding group). Thus, only half of the participants were assumed to intentionally encode and maintain the words in WM in the service of their immediate recall, whereas the other half of the participants were led to believe that the experiment was about juggling different demanding tasks and only incidentally encoded the words. In Experiment 4, the immediate test was manipulated within subjects. Participants silently decided whether the words were living or nonliving things (i.e., an animacy judgment; Experiments 1 and 3–4) or read aloud and pressed the space bar as each word appeared (Experiment 2). As in similar prior work (Oberauer & Greve, 2022; Popov & Dames, 2023), this was to ensure that participants in either encoding group similarly processed the words (e.g., the incidental encoding group did not simply ignore the words altogether). All the participants were surprised at the end of all the experiments with a delayed memory test to assess LTM.

This design allowed us to test the following pre-registered predictions (which can be found on the OSF: https://osf.io/gq2t9/): If the benefits of time are due to actively engaging in some process to keep the information accessible in WM that is also important to LTM, then the previously observed delayed recall advantages of complex span and slow span (i.e., long time) over simple span (i.e., short time) should only occur when participants intentionally encode the words in WM. Conversely, there should be no complex span or slow span advantage to delayed recall when participants only incidentally encode the words given that they would not have actively engaged this key WM process during the free time in-between the words. However, if any time is time well spent simply due to temporal distinctiveness and/or spacing, regardless of any active maintenance, then both intentional and incidental encoding groups should exhibit advantages of complex span and slow span to delayed recall.

The design of Experiments 1–3 necessarily confounds intentionality with immediate testing from WM, with the aim to test these hypotheses *within* the respective intentional and incidental groups. However, to ensure that confounding intentionality and immediate testing did not explain the results of Experiments 1–3, Experiment 4 manipulated the immediate test within the same group of participants. Thus, if the long-term advantage of time spent actively maintaining information in WM is specifically important to LTM, then the benefit of time should be observed regardless of whether the trials end randomly with an immediate test of the words or an irrelevant no-recall task, as in similar prior work (Loaiza et al., 2021; McCabe, 2008).

It is possible that we may observe enhanced delayed performance for the longer time conditions in both of the encoding groups but for different reasons if we assume that LTM reflects a combination of binding memory and item memory. Binding memory involves the retrieval of a studied item and its associated contextual details, such as its original serial position in the trial, whereas item memory refers to memory of the studied items irrespective of their associations or details. This is especially relevant given the theoretical view of WM as a system to establish new bindings and remove outdated and irrelevant ones (Oberauer, 2002, 2009, 2019), as well as broader views that item–context binding is a major factor underlying memory performance (Davelaar et al., 2005; Farrell, 2012; Lehman & Malmberg, 2013; Raaijmakers & Shiffrin, 1980; Unsworth & Engle, 2006, 2007). Thus, participants in the incidental encoding group may exhibit advantages of being presented words in complex span and slow span trials that are driven more strongly by item memory but not binding memory, because the item–position bindings were irrelevant to their task. Conversely, the intentional encoding group may exhibit the advantages in binding memory in addition to item memory given that these item–position bindings were relevant for the WM test. Such a dissociation would suggest that time benefits LTM for two reasons: it may enhance item memory regardless of any active maintenance, but time used intentionally to maintain information in WM may further enhance LTM memory for bindings that were relevant to actively encode and maintain in WM. Using a reconstruction test, wherein participants attempted to select the four presented words in their original order amongst four never-presented lures, allowed us to investigate this possibility by applying hierarchical Bayesian measurement models to estimate binding and item memory in the two encoding groups. Overall, these experiments allow us to directly address whether there is something truly special about active maintenance in WM for the benefit of time for LTM.

## Experiment 1

### Method

#### Participants

We recruited participants via Prolific in Experiments 1, 2, and 4, whereas participants in Experiment 3 comprised a convenience sample of first-year psychology students at the University of Porto. Participants were invited to take part if they were native English (Experiments 1–2, 4) or Portuguese (Experiment 3) speakers, 18–35 years old, reported normal or corrected-to-normal vision, reported no history of cognitive impairment, who were using a desktop/laptop computer, and who had not taken part in any similar prior studies (Loaiza & Lavilla, 2021; Loaiza et al., 2021) or in any of the other experiments of the series (i.e., participants were unique in each experiment). The experiments took 30–40 min for most participants, and they were compensated with £7.50/hour (Experiments 1–2), partial course credit (Experiment 3), or £8/hour (Experiment 4). The project received ethical approval from the University of Essex (Experiments 1 and 2), the University of Porto (Experiment 3), and the University of Sheffield (Experiment 4) Ethical Review Committees. All participants across experiments provided informed consent prior to starting the experiment and were debriefed at the end of the experiment.

Table 1 shows the details of the sample and the exclusion criteria. According to our preregistrations, we aimed to collect data for at least 40 valid datasets per group in each experiment, with a maximum of up to 60 valid datasets per group if necessary to establish the robustness of the findings. This sample size was determined from prior work using the same paradigm (Loaiza & Lavilla, 2021; Loaiza et al., 2021). Participants in Experiments 1–3 were randomly assigned to either the intentional encoding group or the incidental encoding group.^1^ Data from participants were excluded from analysis and replaced if they quit in the middle of the experiment. Data were also excluded and replaced depending on participants’ responses to a final survey (see Materials and Procedure section) for the following reasons: (1) participants who reported experiencing legitimate issues (e.g., technical difficulties) that affected their ability to do the tasks sufficiently; (2) participants who did not conform to their group assignment (i.e., participants in the intentional encoding group who reported that they did not actively try to remember the words; participants in the incidental encoding group who reported that they actively tried to remember the words); and (3) participants who reported that they expected a final memory test. In Experiments 3 and 4, we additionally excluded any participants who quit and restarted the experiment and those who reported using external aids (i.e., cheating, such as taking notes). We conducted the analyses on the full sample following these exclusions as well as on the subsetted data with only the participants who reported complying with the task instructions, the latter of which can be found on the Open Science Framework (OSF).

**Table 1:** Sample details and exclusions

	Exp. 1		Exp. 2		Exp. 3		Exp. 4
Sample details	Int	Inc	Int	Inc	Int	Inc	Int
Total *N* attempted	64	100	63	62	97	85	84
*N* quit at the start/in the middle of the experiment	10	16	12	5	24	16	13
*N* excluded for pre-registered reasons:	11	44	8	13	14	18	10
1. Experienced issues (e.g., technical) during the task*	1	0	2	1	5	6	0
2. Failed to conform to encoding group assignment*	2	42	5	13	4	12	7
3. Expected final memory test*	8	8	3	1	2	0	3
4. Quit and restarted the experiment*†	–	–	–	–	2	0	0
5. Reported cheating*†	–	–	–	–	1	0	0
**Final *N* after preregistered exclusions**	**43**	**40**	**43**	**44**	**59**	**51**	**61**
Further *N* reporting not following instructions	6	9	1	5	10	3	2
Subsetted *N* excluding those not following instructions	37	31	42	39	49	48	59

*Note*. Final sample of the reported analyses is printed in boldface. Exp. = experiment; Int. = intentional encoding; Inc. = incidental encoding. *Note that these may not sum to the total *N* excluded given that participants could report more than one reason that resulted in exclusion. †Note that this exclusion criterion was added for later experiments following further experience with conducting online experiments

#### Materials

The materials for all the experiments can be found on the OSF (https://osf.io/gq2t9/). Experiments 1 and 2 were programmed with Inquisit (Inquisit^5^, ^2018^, and Inquisit^6^, ^2021^, for Experiments 1 and 2, respectively), whereas Experiments 3 and 4 were programmed with lab.js (Henninger et al., 2021) and hosted on Mindprobe, a JATOS server (Lange et al., 2015). For Experiments 1, 2, and 4, a list of 10 practice and 360 critical concrete nouns served as the memoranda, half of which represented living things (letters: *M* = 5.04, *SD* = 1.15, range: 3–7; syllables: *M* = 1.52, *SD* = 0.50, range: 1–2; log HAL frequency: *M* = 8.59, *SD* = 1.28, range: 5.52–12.15; concreteness: *M* = 4.64, *SD* = 0.43, range: 3.00–5.00) and the other half represented nonliving things (letters: *M* = 5.08, *SD* = 1.05, range: 3–7; syllables: *M* = 1.45, *SD* = 0.50, range: 1–2, log HAL frequency: *M* = 8.65, *SD* = 0.93, range: 5.81–10.58; concreteness: *M* = 4.64, *SD* = 0.40, range: 3.07–5.00). For Experiment 3, a similar list of 10 practice and 360 concrete nouns in Portuguese were created, half of which represented living things (letters: *M* = 5.77, *SD* = 2.51, range: 2–11; syllables: *M* = 2.51, *SD* = 0.71, range: 1–5; frequency: *M* = 20.93, *SD* = 33.12, range: 0.06–295.65; concreteness: *M* = 5.99, *SD* = 0.70, range: 3.68–6.81) and the other half represented nonliving things (letters: *M* = 6.10, *SD* = 1.67, range: 2–12; syllables: *M* = 2.62, *SD* = 0.75, range: 1–5; frequency: *M* = 24.31, *SD* = 48.98, range: 0.08–319.36; concreteness: *M* = 6.03, *SD* = 0.74, range: 3.37–6.85).^2^ There were no significant differences between the characteristics of the living and nonliving words in either the English or Portuguese lists, *p*s ≥ 0.055. The experiment drew from these practice and critical lists randomly without replacement during the relevant task for each participant.

#### Procedure

A study advertisement invited participants to take part in an experiment concerning how humans juggle different tasks (Experiments 1–2) or make speeded decisions (Experiments 3–4), with no mention of memory. Participants were advised to be prepared to do the experiment in one continuous sitting in a quiet, distraction-free environment. They were instructed to carefully read and follow instructions, and they were informed that they would be able to view their general performance at the end of (Experiments 1–2) or during (Experiments 3–4) the experiment in order to enhance interest and motivation in the study. After clicking the start link, the experiment filled the screen, thereby preventing participants from engaging in other tasks on their computers during the experiment. Participants then received instructions for and completed three main phases: practice, the immediate task, and the distraction and delayed task. After the final phase, participants completed a final survey and had the chance to view their overall performance on the tasks (Experiments 1–2). The following description of the three main phases is specific to Experiment 1, with the subsequent experiments adjusting details of the task.

##### Practice phase

During the practice phase, participants practiced each relevant element of the immediate task on its own for one block of 10 trials until passing an 85% criterion to move onto the next part. During the words practice, participants viewed a series of words successively presented for 1.5 s (0.1 s interstimulus interval [ISI]).^3^ Participants were instructed to read the words silently and decide whether each word was a living or nonliving thing (i.e., an animacy judgment) using the right- and left-hand arrow keys, respectively. During the arithmetic practice, participants viewed a series of multiplication problems (e.g., 7 × 4 = 30?) presented for 3.5 s (0.1 s ISI) to read aloud and solve whether the answer provided was true or false with the arrow keys. Finally, participants in the incidental encoding group completed practice on the digits task, wherein a series of double-digit numbers (e.g., 23, 48, 51) were successively presented for a self-paced, unfixed duration (0.1 s ISI). Participants were instructed to read the digits silently and decide with the arrow keys whether the digits composing each stimulus were both even (e.g., 42) or not (e.g., 43, 34).

##### Immediate test phase

During the immediate task phase, participants completed trials of simple, complex, and slow span, with the only difference between the encoding groups being how the trials ended: either with a recall attempt (intentional encoding group) or an unrelated digits task (incidental encoding group) as illustrated in Fig. 1A and B. There was one block of 30 trials (10 of each type of task), randomly intermixed, with pauses for breaks after every 10 trials. Participants were informed that for each trial, a fixation (******) would appear at the center of the screen (presented for 1.5 s), followed by four words (each presented for 1.5 s, 0.1 s ISI) to read silently and judge with regard to their animacy. The instructions informed participants that sometimes the four words would appear successively (simple span), whereas during other trials, either an arithmetic problem to read aloud and solve (complex span) or a brief blank screen (slow span) would appear after each word (presented for 3.5 s, 0.1 s ISI). This means that the interval between the offset of one word and the onset of the next was 0.1 s for simple span (short time) and 3.7 s for both the complex span and slow span trials (long time). At the end of the trial, participants in the intentional encoding group saw all four presented words among four never-presented lures, with all eight words randomly arranged in a 2 × 4 grid of frames on the screen. These participants were instructed to use the mouse to select the four presented words in the order that they were presented. For participants in the incidental encoding group, eight double-digit numbers were randomly arranged in a 2 × 4 grid of frames on the screen. These participants were instructed to use the mouse to select the four double-digit numbers that were both even. For both groups of participants, their selections were echoed back to them in a row of four frames at the bottom of the screen. An intertrial interval of 1 s then preceded the start of the next trial.

To ensure that participants stayed on task, they were informed that they must follow these instructions carefully or risk being sent back to the practice round. Specifically, if participants made at least two mistakes on the words or arithmetic problems (i.e., they failed to respond or respond incorrectly), they received an initial warning, after their recall/digit selection but before the next trial began. This warning reminded them of the instructions and informed them that if they continued making mistakes, then they would be sent back to the practice round. If participants then made two mistakes on any subsequent trial after having been warned, they returned to either the words or arithmetic problems practice round, or both, depending on where they fell short. After achieving the 85% criterion, they returned to the main experimental phase, wherein the same warning system continued to apply. Prior work that has implemented this paradigm online has demonstrated that this warning system is effective for motivating compliance with the task instructions in online experiments where an experimenter cannot be present to verify compliance (Loaiza & Lavilla, 2021; Loaiza et al., 2020).

##### Delayed test phase

During the distraction and delayed recall test phase, participants completed 1 min of a symmetry judgment task (taken from Kane et al., 2004; see Loaiza & Lavilla, 2021), which served as a distraction, followed by delayed recall of the words presented in each of the previous trials of the immediate task. During the delayed test, the words of each trial from the previous immediate test were probed in a new random order, wherein the four originally presented words of a given immediate trial were randomly intermixed with four never-presented lures in a 2 × 4 grid of frames. Participants were instructed to recall the words by clicking on the remembered words in their original order, with their responses echoed back to them in a row of frames at the bottom of the screen.^4^

After completing these critical phases, participants completed a final survey wherein they reported basic demographic information (age, gender, native language) and their compliance with the task instructions as well as for their group assignment. Specifically, participants were asked whether they read and answered the arithmetic problems out loud while pressing the keys, whether they read the words silently while making the animacy judgments, whether they actively tried to remember the words when they were first presented,^5^ whether they expected there to be a final memory test at the end, and whether they completed the experiment in one sitting in a quiet distraction-free environment. The survey also asked participants to note any issues arising (e.g., technical) that may have affected their performance. Participants were encouraged to be as honest as possible when answering these questions, with explicit instruction that their answers would not affect their compensation. Responses to these questions were used to justify any exclusions as explained in the Participants subsection.

#### Data analysis

We conducted all preprocessing and analyses in R, using the BayesFactor, brms, and rjags packages (Bürkner, 2018; Morey & Rouder, 2015; Plummer, 2016). We anonymized the participant data by stripping the Prolific IDs (Experiments 1, 2, and 4) and student details (Experiment 3) from the data and replacing them with random ID numbers before putting the raw data on the OSF. All the raw data and scripts for the analyses described further on can be found on the OSF (https://osf.io/gq2t9/).

In general, we relied on Bayesian inference for each of our analyses explained further on, wherein one’s prior beliefs about some parameters of interest (e.g., the effect of task type on retrieval from LTM) are updated in light of the observed data. The updated beliefs are the posterior distributions of these parameters, each of which has a mean and 95% credibility interval (CI) that gives a sense of how certain one can be that the true value of any given parameter lies within that range and whether it overlaps with zero (i.e., a null effect). Furthermore, the means by which one’s prior beliefs are updated is the Bayes factor (BF), which reflects a ratio of evidence for one model over another (e.g., an alternative model assuming an effect of task type over a null model assuming no effect of task type, BF_10_). For greater clarity and comparison between analyses, we will express BFs in favor of the null as their inverse (e.g., a BF_10_ of 0.8 will be expressed as 1/1.25). Like CIs, BFs can be interpreted continuously, such that we can be increasingly convinced by the evidence for or against the null hypothesis as the CI is further away from/more centered on 0 or as the BF increases. Thus, where relevant, we report means [and 95% CIs] for the parameter estimates and/or BFs for one tested model over another.

There were several different measures to assess. First, we detail the performance checks that are not specific to our hypotheses but are nonetheless important: The animacy judgment ensured that participants indeed processed the words similarly between groups, and both groups should be similarly distracted by the arithmetic problems during complex span trials (as reflected by the accuracy of the arithmetic judgments). To ensure that the participants completed these tasks sufficiently and similarly between the two encoding groups, we checked that accuracy during the animacy judgments was similarly high between groups with a 2 (group: intentional, incidental) × 3 (task type: simple, complex, slow) mixed Bayesian analysis of variance (BANOVA), with the default settings. Accuracy on the arithmetic problems during complex span between the two encoding groups was compared with a one-sided Bayesian *t* test. Evidence supporting an absence of any effects or interactions would suggest that the tasks were performed similarly between the two encoding groups and across the different task types. We also verified that participants in the incidental encoding group showed similar accuracy on the double digits task regardless of the type of trial (simple, complex, slow) with a one-way BANOVA. Conversely, a one-way BANOVA and follow-up one-sided Bayesian *t* tests should show that the participants in the intentional encoding group showed the typical immediate performance advantage of simple and slow span over complex span, both in terms of free scoring (i.e., retrieval counted as correct regardless of original serial position) and serial scoring (i.e., retrieval counted as correct only if serial position). For both free and serial scoring, retrieval responses were scored as correct (1) or incorrect (0). This means that chance performance would be 50% for free scoring (4 of 8 items correct) and 12.5% for serial scoring (1 of 8 items correct).

The most important analyses for our predictions pertain to the delayed test. Given that the nature of encoding (intentional vs. incidental) is completely and unavoidably confounded with immediate recall in this experiment, we did not make any comparisons between the two groups of participants for the delayed test, nor was it necessary to compare the groups to address our hypotheses. Instead, we only focus on the contrast of complex and slow span over simple span trials within each of the two groups, both in terms of observed delayed performance and estimated parameters of binding and item memory. For the former, we conducted one-way BANOVA and follow-up Bayesian *t* tests on free and serial scoring, as with immediate recall.^6^ For the sake of brevity, we report these results in tables further on, but we do not discuss them at length given that our hypotheses were more specific to binding and item memory that are derived from fitting models to the performance data, as we discuss next.

**Fig. 2:**
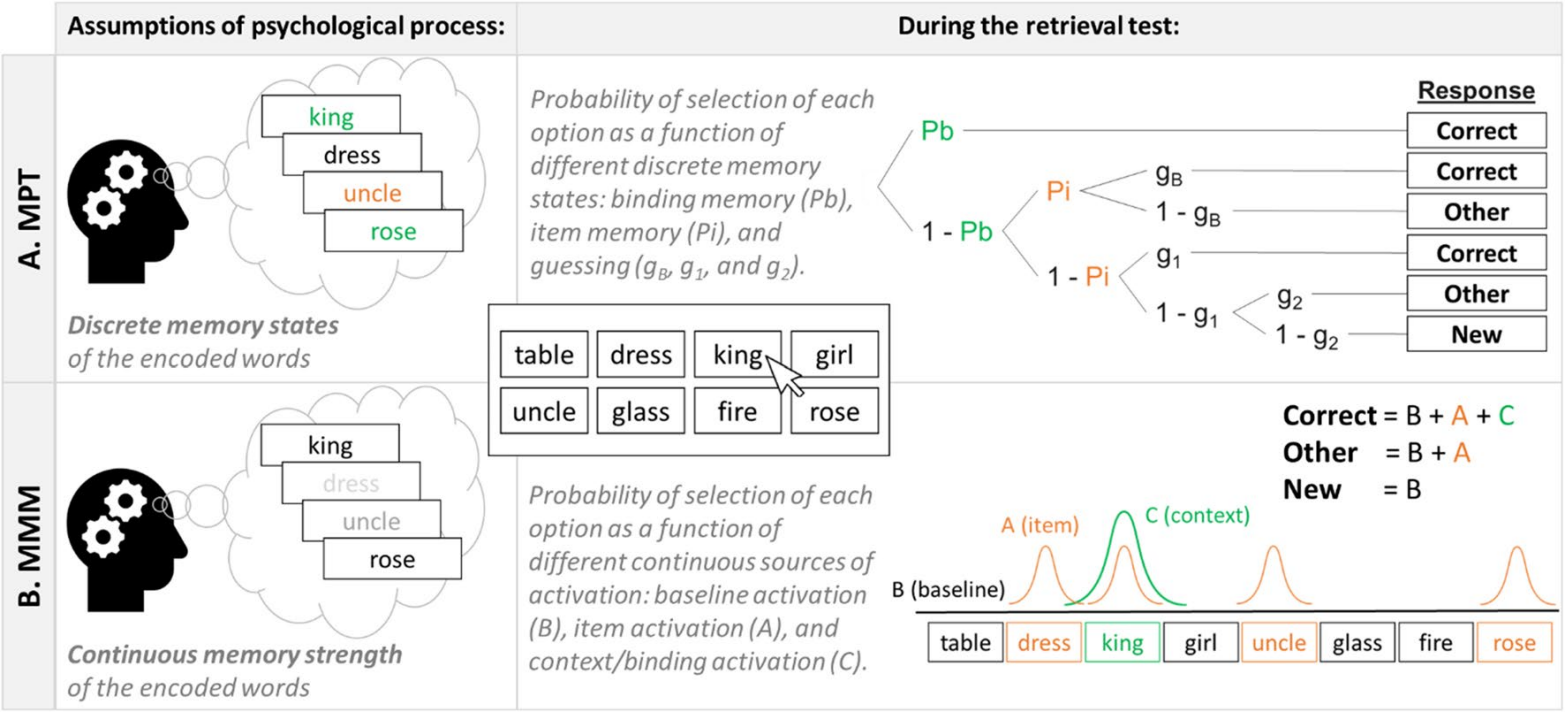
Visual depictions of the multinomial processing tree (MPT) model (**A**) and the memory measurement model (MMM; **B**). (Color figure online)

To estimate parameters of binding and item memory more precisely, we fit both a multinomial processing tree (MPT) model that assumes discrete states of binding and item memory as well as Oberauer and Lewandowsky’s (2019) memory measurement model (MMM) that assumes that these parameters are continuous (Oberauer, 2019). Figure 2 shows a schematic of how each model works. For both models, we followed the same structure and priors as Oberauer (2019) with set size = 4 and response set = 8 (i.e., four studied items and four never-presented lures). Given this, when participants recall from these options for each list position, they can recall either the true target item of that position (Correct), one of the three other presented options (Other), or one of the four never-presented distractors (New).

The MPT model (see Fig. 2A) assumes that recall of the correct target can occur due to accurate binding memory (*Pb*) of the word in its position. In the absence of binding memory (1 − *Pb*), the correct target or one of the other presented items can be guessed with equal probability on the basis of item memory (i.e., memory of the item without its binding, *Pi*). Finally, in the absence of binding memory and item memory (1 − *Pi*), participants guess with equal probability between all the options (see also Bartsch et al., 2019b; Loaiza & Srokova, 2020). The MMM model (see Fig. 2B) assumes that all eight of the response options have a baseline activation *B*, and the four presented items have additional activation *A*, representing item memory. The correct target has an additional activation, *C*, due to the activation of its context that represents binding memory. Note that we truncated the *C* parameter at zero to avoid negative values (Oberauer & Lewandowsky, 2019). The posterior distributions of these parameters were estimated with Bayesian hierarchical implementations of the respective models. We checked that convergence and model fit were adequate, and we drew inferences as previously described for observed performance. The results of both the MMM and MPT models can be found on the OSF, but for the sake of brevity, we report the results of only the MMM here which were broadly consistent with the MPT results except where noted.

### Results and discussion

#### Auxiliary task performance

As explained previously, we first assessed performance on the auxiliary tasks (i.e., accuracy of the animacy word-processing task, accuracy of arithmetic problems during complex span, digit accuracy in the incidental encoding group, immediate memory performance in the intentional encoding group, and delayed memory performance in both groups; see Table 2), which do not pertain to the hypotheses but still allow us to draw firmer conclusions about them. To briefly summarize this section, both encoding groups showed similarly high accuracy when making animacy decisions and responding to the arithmetic problems, and complex span reduced immediate recall performance in the intentional encoding group. The evidence for differences between task types in delayed free and serial scoring performance was relatively weak.

**Table 2:** Means (and standard deviations) of proportion accuracy on the auxiliary and recall tasks in Experiments 1 and 2

		Experiment 1 Animacy judgements		Experiment 2 Read and press space bar	
Measure	Task type	Intentional	Incidental	Intentional	Incidental
Word-processing accuracy	simple	0.91 (0.07)	0.91 (0.07)	0.98 (0.02)	0.99 (0.02)
	complex	0.81 (0.10)	0.84 (0.11)	0.94 (0.07)	0.96 (0.06)
	slow	0.91 (0.06)	0.91 (0.06)	0.98 (0.03)	0.98 (0.05)
Read-aloud accuracy	simple	–	–	0.79 (0.35)	0.76 (0.35)
	complex	–	–	0.77 (0.34)	0.75 (0.36)
	slow	–	–	0.78 (0.35)	0.74 (0.38)
Arithmetic problems accuracy	complex	0.90 (0.07)	0.89 (0.08)	0.91 (0.08)	0.91 (0.08)
Digit task accuracy	simple	–	0.98 (0.03)	–	0.98 (0.04)
	complex	–	0.98 (0.03)	–	0.97 (0.04)
	slow	–	0.98 (0.02)	–	0.97 (0.04)
Immediate recall—free scoring	simple	0.99 (0.02)	–	0.99 (0.02)	–
	complex	0.95 (0.05)	–	0.94 (0.05)	–
	slow	0.98 (0.04)	–	0.99 (0.02)	–
Immediate recall—serial scoring	simple	0.90 (0.10)	–	0.92 (0.07)	–
	complex	0.71 (0.19)	–	0.65 (0.18)	–
	slow	0.91 (0.11)	–	0.92 (0.09)	–
Delayed recall—free scoring	simple	0.85 (0.09)	0.76 (0.11)	0.75 (0.12)	0.67 (0.11)
	complex	0.86 (0.09)	0.77 (0.09)	0.77 (0.13)	0.64 (0.10)
	slow	0.87 (0.10)	0.79 (0.11)	0.77 (0.13)	0.67 (0.12)
Delayed recall—serial scoring	simple	0.39 (0.17)	0.22 (0.10)	0.28 (0.15)	0.19 (0.09)
	complex	0.42 (0.17)	0.20 (0.07)	0.29 (0.13)	0.16 (0.06)
	slow	0.45 (0.20)	0.23 (0.10)	0.37 (0.16)	0.18 (0.07)

For the word-processing task accuracy, a 2 (encoding group: intentional, incidental) × 3 (task type: simple, complex, slow) mixed BANOVA showed strong evidence for only an effect of task type (BF_10_ = 3.99e16) that was substantially preferred (BF = 3.22) to the next best model that included a further effect of encoding group (BF_10_ = 1.24e16). Thus, animacy decisions during word-presentation were less accurate overall during complex span compared with simple and slow span, but this did not vary by encoding group. Next, a one-sided Bayesian *t* test showed strong evidence against the effect of encoding group on accuracy during the arithmetic problems of complex span (BF_10_ = 1/13.58). Thus, both encoding groups were similarly engaged with processing the words as they were presented as well as with the distraction task that interspaced the words in complex span trials, regardless of their different encoding instructions.

Next, a one-way BANOVA on accuracy to select the correct double-digit numbers at the end of the trials for the incidental encoding group showed strong evidence against an effect of task type (BF_10_ = 1/7.09). We further assessed immediate memory performance in the intentional encoding group in terms of both free and serial scoring with respective one-way BANOVAs. Both analyses showed strong evidence for an effect of task type (free: BF_10_ = 48,733; serial: BF_10_ = 6.34e13), such that correct word reconstruction from complex span trials was considerably lower than both simple span (free: BF_10_ = 2,398; serial: BF_10_ = 3.71e7) and slow span (free: BF_10_ = 1,411; serial: BF_10_ = 9.39e8). However, there was little evidence of a difference in performance between simple and slow span (free: BF_10_ = 1/2.10; serial: BF_10_ = 1/11.12). Thus, the typical disadvantage to immediate memory from complex span relative to simple and slow span was observed in the intentional encoding group, whereas task type had no effect on the incidental encoding group’s irrelevant task, as expected. It is important to establish these results to ensure that the next most important results pertaining to the central hypotheses could not be due to differences between the encoding groups in these auxiliary tasks.

Finally, regarding observed delayed serial and free scoring, one-way BANOVAs and follow-up Bayesian *t* tests showed weak evidence for a benefit of slow span over simple span in serial scoring of the intentional encoding group (BF_10_ = 2.38), and weak to moderate evidence against any differences between task types for either group and for either free or serial scoring (BF_10_s ranging from 1.00 to 1/5.73). However, free and serial scoring are not process-pure measures of item and binding memory, respectively, and thus the next reported analyses allowed us to estimate these parameters more precisely.

#### Parameter estimates of item and binding memory

**Fig. 3:**
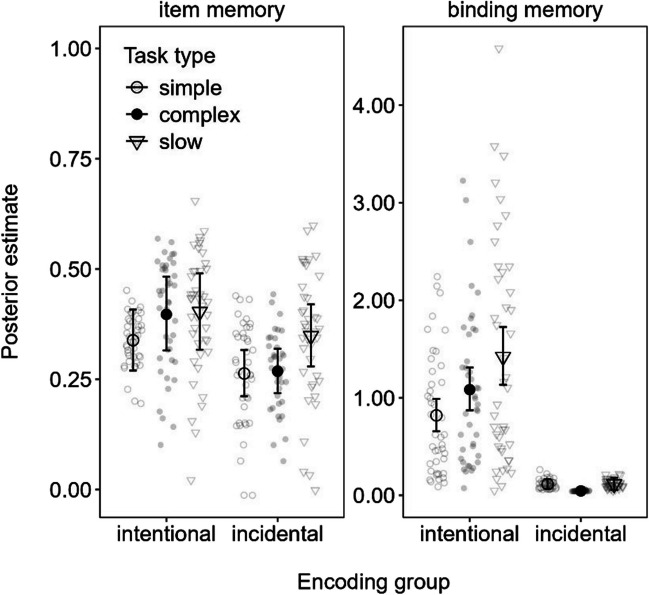
Mean parameter estimates from the memory measurement model (MMM) fitted to the delayed recall data in Experiment 1. *Note.* Error bars reflect 95% credibility intervals, and individual points reflect posterior predicted responses based on the models

The item memory and binding memory parameters estimated from the MMM model are shown in Fig. 3. The intentional encoding group showed a clear slow span advantage over simple span in binding memory (0.60 [0.26, 0.95]), whereas there were no task type differences in item memory. For the incidental encoding group, there was a weak negative effect of complex span relative to simple span in binding memory (− 0.07 [− 0.13, − 0.01]). Given the fact that binding memory was at floor in the incidental encoding group, we abstain from interpreting this effect. Regarding item memory, there was just-barely credible advantage of slow span over simple span in the incidental encoding group according to the MPT model (0.05 [0.00, 0.09]; see results in the OSF), but this difference was not credible in the MMM (0.09 [0.00, 0.17]) shown in Fig. 3.

In sum, actively maintaining information in WM during slow span selectively increased binding memory for the intentional encoding group, whereas negligible differences between tasks were observed for binding memory for the incidental group. Overall, the dissociation is congruent with our preregistered suggestion that time may benefit episodic LTM when it is used intentionally to maintain bindings that were relevant to actively encode and maintain in WM. Furthermore, the fact that no such advantage was consistently observed for complex span suggests that uninterrupted free time following each word is most important to strengthening these different benefits of time on LTM.

## Experiment 2

The results of Experiment 1 indicated that the benefits of actively maintaining information in WM over uninterrupted time specifically reinforce the long-term retention of bindings. However, the relatively smaller effects and the fact that the time advantage was specific to slow span is somewhat inconsistent with prior work using this paradigm which Experiment 1 closely reproduced (Loaiza & Lavilla, 2021; Souza & Oberauer, 2017). Indeed, fitting the aforementioned models to the data of Loaiza and Lavilla (2021; Experiment 2) showed clearer advantages of complex span and slow span over simple span to binding memory, both when participants reported using spontaneous elaborative strategies (e.g., imagery, grouping; MMM: complex span = 0.59 [0.20, 0.98]; slow span = 1.90 [1.24, 2.60]) and ineffective strategies (e.g., rehearsal, reading; MMM: complex span = 0.21 [0.14, 0.28]; slow span = 0.35 [0.26, 0.44]; see OSF for further details).

One potential procedure difference that may explain this discrepancy is that participants were required to make a silent animacy judgment during word presentation in Experiment 1, whereas prior work asked participants to simply read the words aloud. As in other similar research manipulating intentional encoding (Oberauer & Greve, 2022; Popov & Dames, 2023), the animacy judgment of Experiment 1 was only intended to ensure that the intentional and incidental encoding groups similarly attended to the words. However, this may have inadvertently engendered an elaboration effect across the three types of trials (namely, simple, complex, and slow span) that was strong enough to outweigh the benefits of longer maintenance time in WM. Thus, while the point of the experiment was not to investigate elaboration, our results indicated that an enforced elaboration during word-processing may have minimized the long-term benefit of time in WM. This may suggest that elaboration is a crucial process underlying this benefit, but the answer to our principal research question is still unclear regarding whether whatever process engaged (perhaps elaboration or something else) must be in service to keeping information active in mind to observe the long-term benefits of time in WM.

To address this lingering issue, we conducted a second experiment that was very similar to our first experiment, except that participants were instructed to read the presented words aloud and press the space bar when they finished reading (so that we could monitor online performance). This was more similar to prior work that only requires participants to read the words aloud during the WM task (e.g., Loaiza & Lavilla, 2021; Souza & Oberauer, 2017), while also ensuring that participants attended to the words regardless of their encoding group; the program returned participants to the practice round for failing to follow these instructions (i.e., to press the space bar). Moreover, participants’ voice responses were recorded and transcribed offline after data collection was completed to verify that both encoding groups similarly followed these instructions.

Our predictions were the same as in Experiment 1: If actively engaging in some maintenance process underlies the benefit of time for LTM, then the delayed advantages of complex span and slow span over simple span should be unique to when participants intentionally encode the items in WM, whereas no such advantages should be observed under incidental encoding conditions. However, if active maintenance in WM is not necessary for these long-term benefits, then both the intentional and incidental encoding groups should exhibit delayed advantages of complex span and slow span over simple span. Furthermore, as previously explained, it may be possible that these advantages may differently occur for the two encoding groups if active maintenance in WM is particularly important for binding memory than item memory. Thus, the delayed advantages of complex span and slow span may occur only in item memory for the incidental encoding group, whereas the intentional encoding group may show these advantages in both binding and item memory.

### Method

#### Materials, procedure, and data analysis

The experiment materials, procedure, and data analysis methods were identical to Experiment 1, with the main exception being that participants were instructed to read the presented words out loud and press the space bar when they finished but before the word disappeared from the screen. Participants practiced this task for 10 trials before the critical phase of the experiment, and they were instructed to read at a normal rate and to not press the space bar too quickly. The experiment only registered responses 0.2 s after stimulus onset, and participants were warned that responding too quickly or failing to respond would count as an error. As in Experiment 1, the words were presented each for 1.5 s (0.1 s ISI), and participants were warned that they would repeat the practice if they made too many errors to motivate them to stay on task. Participants’ voice responses during the presentation of the words and the arithmetic problems were recorded, and a trained research assistant transcribed the audio files for all participants who completed the experiment for offline compliance check.^7^

One further minor change was to include additional instruction screens to facilitate administration of the experiment: A screen at the start of the experiment instructed participants on how to arrange their hands to avoid switch costs between the space bar and the arrow keys during the words and arithmetic problem tasks, respectively. Additional instruction screens began each of the main phases of the experiment (i.e., practice, critical task, and distraction/delayed recall) to clearly demarcate the elements of the experiment to the participants for later ease of reference during the final survey.

### Results and discussion

#### Auxiliary task performance

As in Experiment 1, we first report performance on the auxiliary tasks that did not concern the hypotheses but still are important to verify: Accuracy of pressing the space bar, read-aloud accuracy, accuracy of arithmetic problems during complex span, digit accuracy in the incidental encoding group, immediate recall performance in the intentional encoding group, and delayed recall performance in both groups (see Table 2). To briefly summarize, the results were consistent with Experiment 1: Both encoding groups were similarly engaged, regardless of task type. Immediate performance in the intentional group was unsurprisingly worse for complex span compared with simple and slow span. Delayed performance was generally greater for slow span than simple and complex span in the intentional but not incidental encoding group.

Regarding accuracy to press the space bar during word-processing, a 2 (encoding group: intentional, incidental) × 3 (task type: simple, complex, slow) mixed BANOVA showed strong evidence for only an effect of task type (BF_10_ = 963,735) that was substantially preferred (BF = 3.68) to the next best model that included a further effect of encoding group (BF_10_ = 261,939). Thus, accuracy during the space bar task was worse for complex span compared with simple and slow span, but this did not vary by encoding group. The same 2 (encoding group) × 3 (task type) mixed BANOVA on read-aloud accuracy showed ambiguous evidence against an effect of encoding group (BF_10_ = 1/1.55) and strong evidence against the remaining main effect and interaction models (all BF_10_s > 1/16.27). A one-sided Bayesian *t* test also showed strong evidence against an effect of encoding group on accuracy during the arithmetic problems of complex span (BF_10_ = 1/8.07). Thus, both encoding groups were similarly compliant with instructions to orient to the words by saying them aloud and pressing the space bar, as well as similarly engaged with the distraction of complex span, regardless of their different encoding instructions.

Next, a one-way BANOVA on accuracy to select the correct double-digit numbers at the end of the trials for the incidental encoding group showed substantial evidence against an effect of task type (BF_10_ = 1/4.06). We also assessed immediate memory performance in the intentional encoding group in terms of both free and serial scoring with respective one-way BANOVAs. Both analyses showed strong evidence for an effect of task type (free: BF_10_ = 7.26e10; serial: BF_10_ = 8.28e22), such that performance from complex span was considerably lower than both simple span (free: BF_10_ = 482,453; serial: BF_10_ = 6.17e11) and slow span (free: BF_10_ = 212,269; serial: BF_10_ = 4.02e11). However, there was no difference in performance between simple and slow span (free: BF_10_ = 1/3.57; serial: BF_10_ = 1/7.58). Thus, the typical complex span disadvantage to immediate memory was observed in the intentional encoding group, whereas task type had no effect on the incidental encoding group’s irrelevant task, as expected. As in Experiment 1, these results indicate that the next most important results pertaining to the central hypotheses could not be due to differences between the encoding groups in these auxiliary tasks.

Finally, regarding observed delayed performance, one-way BANOVAs and follow-up Bayesian *t* tests of serial scoring showed substantial evidence for a benefit of slow span over simple span (BF_10_ = 77.05) and complex span (BF_10_ = 47.44) in the intentional encoding group, and ambiguous to moderate evidence against any differences between simple and complex span in serial scoring or any task type differences in free scoring (BF_10_s ranging from 1.01 to 1/6.00). For the incidental group, slow span yielded greater delayed serial scoring than complex span (BF_10_ = 6.34), with no further differences between task types for either free or serial scoring (BF_10_s ranging from 1.43 to 1/6.00). As free and serial scoring cannot be considered as process-pure measures of item and binding memory, the next analyses allowed us to consider these parameters more precisely.

#### Parameter estimates of item and binding memory

**Fig. 4:**
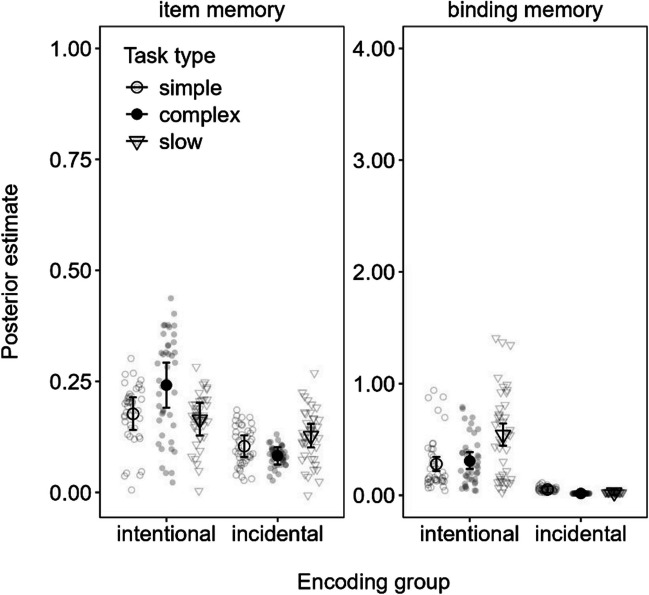
Mean parameter estimates from the memory measurement model (MMM) fitted to the delayed recall data in Experiment 2. *Note.* Error bars reflect 95% credibility intervals and individual points reflect posterior predicted responses based on the models

The item memory and binding memory parameters estimated from the MMM model are shown in Fig. 4. The intentional encoding group showed a clear advantage to binding memory for slow span over simple span (0.26 [0.15, 0.38]) as well as over complex span (0.23 [0.11, 0.36]). This replicates the benefit of free time for binding memory observed in Experiment 1, in line with our main hypothesis. The incidental group showed no effect on binding memory, except for a barely credible disadvantage to binding memory of complex span versus simple span (− 0.04 [− 0.07, 0.00]) for the MMM model depicted in Fig. 4. We note, however, that this effect was not credible in the MPT model (see OSF), which further reduces the reliability of this effect.

Furthermore, we observed better item memory in complex span compared with slow span in the intentional encoding group (0.08 [0.01, 0.14]), but worse item memory for complex span compared with slow span (− 0.04 [− 0.08, − 0.01]) in the incidental encoding group. These results were rather inconsistent and weak, and hence we abstain from interpreting them.

The main results of Experiment 2 complement and extend those of Experiment 1: Actively maintaining information in WM during slow span improved the long-term retention of bindings (i.e., intentional encoding group). Notwithstanding, comparing Figs. 3 and 4 shows overall greater item and binding memory in Experiment 1 compared with Experiment 2 wherein participants were not required to make elaborative decisions on the presented words. These observations are in line with previous reports of elaboration benefits in LTM.

Overall, these results suggest that longer time may differently benefit underlying parameter estimates of binding memory in LTM depending on whether participants actively maintained the information in WM. Binding benefits were consistently observed for slow span trials in the intentional encoding group across both experiments.

## Experiment 3

The results thus far consistently show a slow span effect in binding memory during retrieval from episodic LTM (i.e., an advantage of intentionally studying words during slow span over simple span and complex span). This suggests that active maintenance during uninterrupted free time in WM is particularly important to the retention of bindings in the long term.

What remains unclear is the mechanism that is responsible for this effect. It is unlikely that elaboration is exclusively responsible given that the effect was observed regardless of whether participants made elaborative decisions on the memoranda (Experiment 1) or not (Experiment 2). Similarly, Loaiza and Lavilla (2021) showed that a slow span advantage was observed regardless of whether spontaneous or instructed strategies were elaborative (e.g., imagery, grouping) or ineffective (e.g., rehearsal, reading). Thus, elaboration clearly enhances retrieval from LTM overall, but it is unlikely to be the sole underlying cause of the benefit of free time in WM. It may be the case that affording more time for active maintenance helps to recover depleted encoding resources that are important to establishing and retaining bindings in WM, consistent with Popov and Reder’s (2020) resource model. Alternatively, more time in WM may allow a short-term consolidation process to stabilize the binding between the item and its context (Cotton & Ricker, 2021).

To this end, the next experiment in the series replicated the design of Experiment 1, except that it replaced the complex span condition with a “medium” free-time condition alongside the existing short (simple span) and long (slow span) free-time conditions. This modification was done to establish whether increasing the time following each item results in a corresponding increase in binding memory. Accordingly, we expected to replicate the main result of Experiment 1, such that increasing the uninterrupted free time to intentionally encode words in WM results in corresponding increases in later binding memory. Furthermore, Experiment 3 provided the opportunity to assess if we could replicate the relatively weak finding of Experiment 1 that increasing the free time under incidental encoding increases item memory.

### Method

#### Materials and procedure

The experiment materials and procedure were identical to Experiment 1, with the main exception that the instructions and stimuli were presented in Portuguese as well as replacing the complex span condition with a “medium” free-time condition (1.9 s ISI following each word) alongside the short (simple span; 0.1 s ISI) and long (slow span; 3.7 s ISI) free-time conditions. Participants also received feedback about their general performance during each break rather than at the end of the study.

### Results and discussion

#### Auxiliary task performance

The results of the auxiliary tasks are presented in Table 3. To summarize, as in the previous experiments, the intentional and incidental encoding groups showed similarly high accuracy on the animacy word-processing task, and there was little evidence of an effect of task on performance during the no-recall digit task (incidental group) or free scoring of the immediate recall test (intentional group). Conversely, immediate serial and delayed performance were greater for long versus short time intervals in the intentional group, and there was no evidence of differences in delayed performance in the incidental group.

**Table 3:** Means (and standard deviations) of proportion accuracy on the auxiliary and recall tasks in Experiments 3 and 4

		Experiment 3		Experiment 4	
Measure	Time	Intentional	Incidental	Serial recall	No recall
Word-processing accuracy	short	0.92 (0.05)	0.94 (0.04)	0.95 (0.04)	0.93 (0.06)
	medium	0.94 (0.05)	0.95 (0.04)	–	–
	long	0.93 (0.05)	0.94 (0.04)	0.94 (0.05)	0.94 (0.07)
Digit task accuracy	short	–	0.96 (0.06)	–	0.97 (0.06)
	medium	–	0.96 (0.05)	–	–
	long	–	0.97 (0.04)	–	0.97 (0.05)
Immediate recall—free scoring	short	0.97 (0.03)	–	0.97 (0.04)	–
	medium	0.97 (0.03)	–	–	–
	long	0.98 (0.03)	–	0.99 (0.02)	–
Immediate recall—serial scoring	short	0.85 (0.13)	–	0.83 (0.18)	–
	medium	0.89 (0.12)	–	–	–
	long	0.91 (0.11)	–	0.86 (0.19)	–
Delayed recall—free scoring	short	0.79 (0.13)	0.78 (0.10)	0.80 (0.12)	0.74 (0.11)
	medium	0.81 (0.13)	0.80 (0.10)	–	–
	long	0.82 (0.13)	0.80 (0.10)	0.82 (0.11)	0.78 (0.12)
Delayed recall—serial scoring	short	0.31 (0.16)	0.25 (0.11)	0.33 (0.18)	0.30 (0.15)
	medium	0.35 (0.18)	0.27 (0.12)	–	–
	long	0.38 (0.19)	0.23 (0.10)	0.37 (0.20)	0.34 (0.18)

More specifically, a 2 (group) × 3 (time) mixed BANOVA on proportion accuracy during the word-processing task showed ambiguous evidence for a main effect of encoding group (BF_10_ = 1.35) and evidence against the other main effects and interaction models (BF_10_s ranging 1/4.79 to 1/45). A one-way BANOVA on digit task accuracy in the incidental encoding group showed ambiguous evidence against an effect of time on performance (BF_10_ = 1/1.28). Similarly, there was negligible evidence for a main effect of time on immediate free scoring in the intentional encoding group (BF_10_ = 1/2.06). However, the same one-way BANOVA on immediate serial scoring in the intentional encoding group showed strong evidence for an effect of task (BF_10_ = 48.08), with follow-up Bayesian *t* tests showing that the difference between short versus long conditions largely driving this effect (BF_10_ = 113.40). Finally, delayed free and serial scoring mirrored performance on the immediate test in the intentional group, such that the overall effect of task type (free: BF_10_ = 11.27, serial: BF_10_ = 14.99) was largely driven by a difference between the short and long time intervals (free: BF_10_ = 21.24, serial: BF_10_ = 13.42), whereas there was mostly ambiguous evidence for differences between the other time intervals (BF_10_s ranging 2.42 to 1/2.97). Finally, corresponding one-way BANOVAs in the incidental encoding group showed ambiguous evidence against a main effect of time on delayed performance (free: BF_10_ = 1/1.42, serial: BF_10_ = 1/1.86). As in the previous experiments, the purer parameter estimates of binding and item memory were most crucial to our hypotheses, which we turn to in the next section.

#### Parameter estimates of item and binding memory

**Fig. 5:**
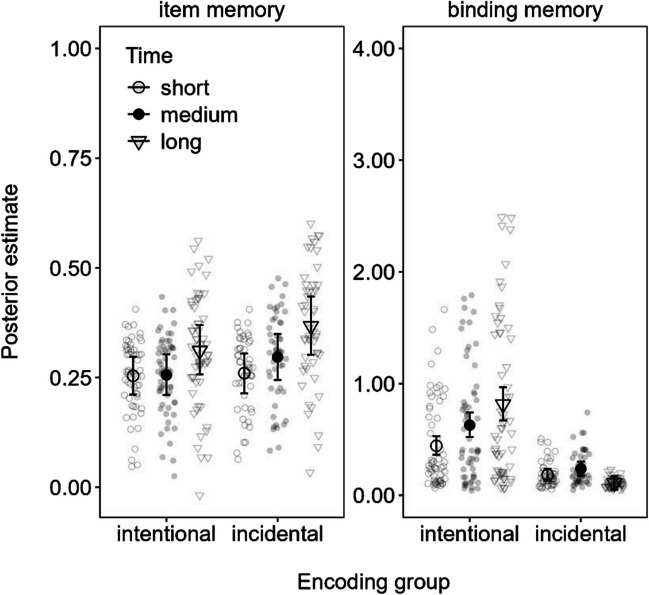
Mean parameter estimates from the memory measurement model (MMM) fitted to the delayed recall data in Experiment 3. *Note.* Error bars reflect 95% credibility intervals and individual points reflect posterior predicted responses based on the models

The parameter estimates of item and binding memory from the MMM model in Experiment 3 are presented in Fig. 5. As in the previous experiments, there was a clear binding memory advantage for long over short time intervals (0.37 [0.20, 0.55]) in the intentional encoding group, as well as a benefit of medium over short periods of time (0.19 [0.05, 0.32]). The difference between long- and medium-time intervals was just barely credible in the MMM (0.19 [0.00, 0.37]), but we should note that this was not credible in the MPT model (available on the OSF). The incidental encoding group showed little consistent effects of time on binding memory, except for credibly worse binding in the long versus medium time condition (− 0.13 [− 0.21, − 0.04]). Again, binding memory was generally low for this group, and hence we refrain from interpreting these results.

For item memory, the intentional encoding group showed no credible effects of time, whereas the incidental encoding group showed a credible advantage of long versus short time intervals to item memory (0.11 [0.03, 0.19]).

In line with the previous experiments and our hypotheses, increasing the time for actively maintaining information in WM resulted in corresponding increases to later binding memory in the intentional encoding group. This pattern was not evident in the incidental encoding group, which instead showed greater item memory for longer versus shorter time intervals. This latter result is partly in line with the results of Experiment 1, which suggested weak evidence for a similar difference in the MPT model but not the MMM. The evidence for this benefit of time to item memory in the incidental encoding group may have been stronger in Experiment 3 given the slightly greater number of participants than in Experiment 1. Notwithstanding, the pattern will need to be replicated in future experiments to be certain. We return to this dissociation of binding and item memory on the basis of encoding group in the General Discussion.

## Experiment 4

In our previous experiments, we observed that the intentional retention of information in WM for a longer free-time period promoted long-term learning of binding information compared with an incidental condition. The aim of Experiment 4 was to address whether the results of the previous experiments could be explained by a testing effect. That is, the intentional encoding group engaged in retrieval of the items during the immediate test of WM, whereas the incidental encoding group did not, and thus intentionality and testing are necessarily confounded, as we attested to earlier on. We tried to mitigate this issue by not comparing the intentional and incidental encoding groups directly in any analysis, but it still leaves the possibility that the different patterns of results between the encoding groups can be explained by a mere effect of retrieval practice rather than intentionality, as was our aim.

Although retrieval practice is likely to enhance overall performance, it is unlikely to explain the observed benefit of free time given similar prior work showing a delayed advantage of complex span (longer time) over simple span (shorter time) regardless of whether the trial ended randomly in an immediate test of the items or an irrelevant task (Loaiza et al., 2021, Experiment 3; McCabe, 2008, Experiment 3). That is, the act of engaging in active maintenance in WM over a longer versus shorter period of time was responsible for the advantage, although immediate testing enhanced performance overall for both conditions. However, to be sure that is the case here as well, this experiment aimed to replicate this result following the methods from the previous experiments of this series to make it easier to compare between them.

Specifically, participants were instructed to engage in intentional encoding of the items in trials with short and long free time (manipulated via the ISIs, as in the previous experiments). To assess the impact of testing, half of the trials in each time condition ended randomly with either a serial-recall or no-recall parity task. Thereafter, participants completed a surprise test to assess episodic LTM, as in the previous experiments. If the immediate task does indeed impact the importance of uninterrupted maintenance time in WM for LTM trace formation, then the delayed advantage of free time should be reduced or nullified when trials end with a no-recall parity task compared with an immediate serial-recall task (i.e., a Time × Immediate Task interaction). However, if active maintenance in WM drives the effects we have observed, then the free-time effect should be evident and similar for both types of immediate task, although there is likely to be an overall benefit of serial recall over no recall (i.e., main effects of time and immediate task, but no interaction). Unlike the previous experiments, this can be assessed directly by comparing the free-time effects within both immediate tests. Furthermore, we considered these effects both in overall delayed performance as well as the measures of binding and item memory derived from fitting the same models of the previous experiments to the data. Thus, we expected to find that binding memory is particularly strengthened with time, regardless of immediate testing.

### Method

#### Materials and procedure

The materials and procedure were similar to the previous experiments, with the main exceptions that there were only short (simple span, 0.1 s ISI) and long (slow span, 3.7 s ISI) free-time conditions, and half of each ending randomly in either the serial-recall or no-recall parity task. Thus, all participants in the experiment were instructed to silently study and try to remember the four sequentially presented words while making an animacy decision for each. They were further instructed that some of the trials would end randomly with an immediate test of their memory or a digit task. There were nine trials per cell of the design (i.e., 36 trials total, randomly intermixed for each participant).^8^ Breaks were provided after every 12 trials, during which overall performance was presented as in Experiment 3. The memoranda were the same English words as in Experiments 1 and 2.

### Results and discussion

#### Auxiliary task performance

The results of the auxiliary tasks are presented in Table 3. To summarize, as in the previous experiments, there was little evidence that participants differently engaged with the word-processing task (i.e., animacy decisions during encoding) or digit parity task during the no-recall trials depending on the independent variables of the experiment, but longer free time benefitted free and immediate serial scoring during the trials requiring serial recall. These benefits of free time extended to delayed free and serial scoring, with further evidence for a main effect of immediate test on free scoring.

Specifically, 2 (time: short, long) × 2 (immediate test: serial recall, no recall) within-subjects BANOVA showed little evidence for models assuming any main effects or interaction on accuracy during the word-processing task (BF_10_s ranging 1/2.87 to 1/19.27). Furthermore, there was substantial evidence against an effect of time on digit task accuracy during the no-recall trials (BF_10_ = 1/4.63), but substantial evidence for an effect of time on immediate performance during the serial-recall trials (free: BF_10_ = 12.23, serial: BF_10_ = 3.08). Finally, a 2 (time) × 2 (immediate test) within-subjects BANOVAs were conducted on delayed free and serial scoring. The best model for free scoring included main effects of time and immediate test (BF_10_ = 1.15e9) that was anecdotally preferred (BF = 1.51) to the next best model including both main effects and an interaction (BF_10_ = 7.61e8). For serial scoring, the best model included a main effect of time (BF_10_ = 5.52) that was anecdotally preferred (BF = 1.29) to the next best model including both main effects (BF_10_ = 4.29). As in the previous experiments, our most important analyses pertained to the parameter estimates of item and binding memory.

#### Parameter estimates of item and binding memory

**Fig. 6:**
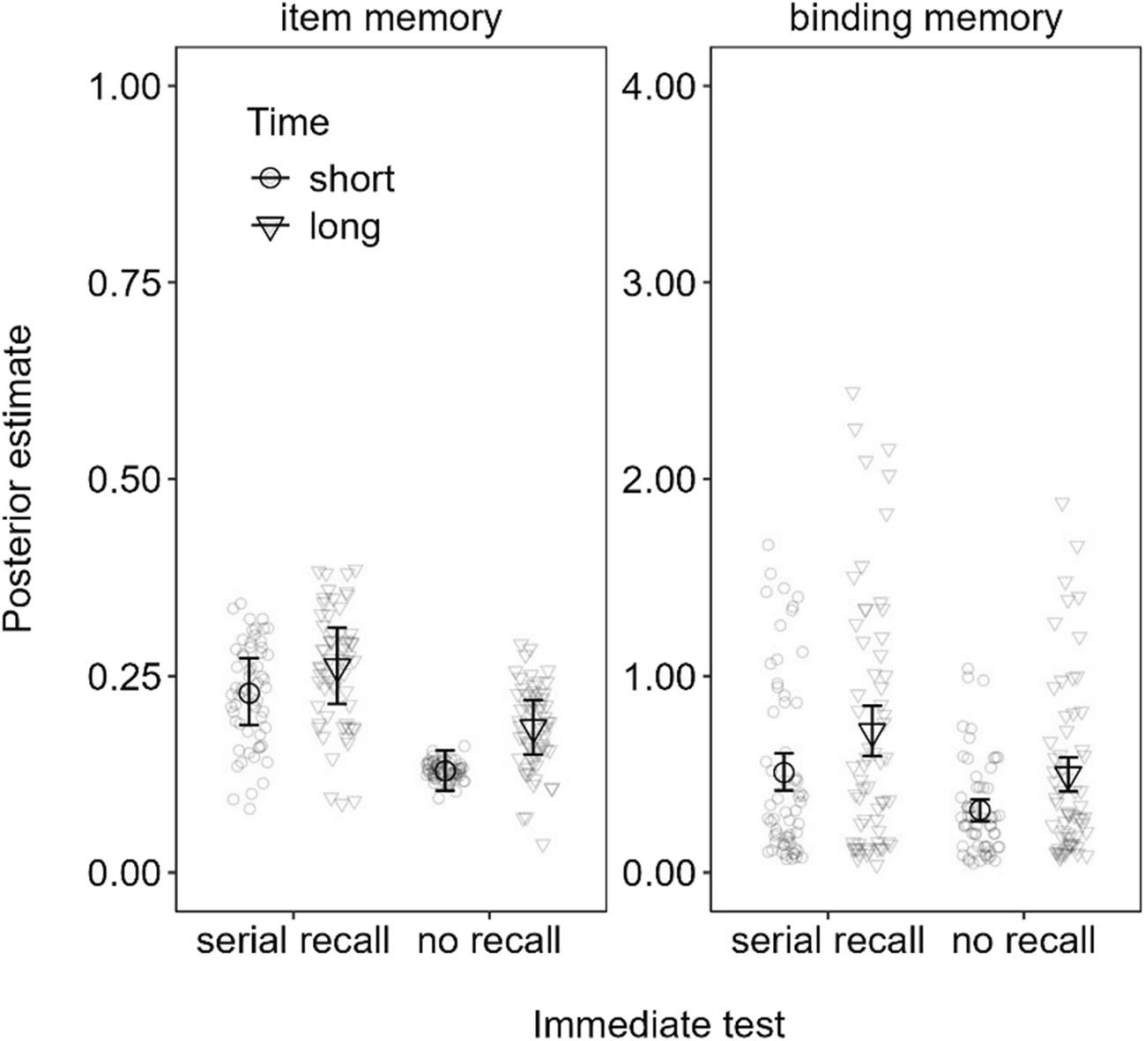
Mean parameter estimates from the memory measurement model (MMM) fitted to the delayed recall data in Experiment 4. *Note.* Error bars reflect 95% credibility intervals and individual points reflect posterior predicted responses based on the models

Figure 6 shows the parameter estimates of item and binding memory from Experiment 4. Consistent with the previous experiments and our predictions, binding memory was credibly greater for serial-recall trials with a long versus short ISIs (0.21 [0.05, 0.37]). Importantly, this was also the case for no-recall trials (0.18 [0.07, 0.28]), in line with our hypothesis that active maintenance in WM is uniquely important to the free-time advantage, rather than attributable to a mere testing effect, which was a possible alternative explanation of the prior results.

Finally, there was also a small but credible advantage of longer free time to item memory during the no-recall trials (0.06 [0.01, 0.10]) but not the serial-recall trials (0.03 [− 0.03, 0.10]). This dissociation is somewhat reminiscent of the previous indication that uninterrupted free time for active maintenance is particularly important to binding memory, but free time can also be advantageous to item memory when either incidentally encoding the information (Experiment 3) or when it was not immediately recalled (as here in Experiment 4). Again, this finding will need to be replicated in the future, and we return to this possibility in the General Discussion.

## General discussion

The current experiments tested whether there is something truly special about active maintenance in WM for the benefit of time on episodic LTM. The first two experiments achieved this by encouraging either the intentional or incidental encoding of memoranda that were either presented successively following a simple span procedure (i.e., short time interval) or each followed by a period of interrupted or uninterrupted time following a complex span and slow span procedure, respectively (i.e., longer time interval). Furthermore, Experiment 3 varied the amount of uninterrupted free time (short, medium, long), and Experiment 4 manipulated immediate test (serial or no recall) within-subjects rather than between-subjects as in the previous experiments. If the benefits of time on episodic LTM depend on processes carried out during WM maintenance, then longer time intervals should produce better LTM than short time only when the memoranda were actively encoded and maintained in WM (i.e., participants in the intentional encoding groups across all four experiments). However, if the benefits of time occur due to mere effects of spacing or temporal distinctiveness, then long-term advantages of longer time intervals should be observed regardless of active maintenance in WM (i.e., for intentional and incidental encoding groups in Experiments 1–3). Furthermore, if the observed benefits of time in the intentional encoding group were due to a mere testing effect, then the benefit should be reduced or nullified during no-recall trials in Experiment 4, wherein all the participants were engaged in intentional encoding but whose trials randomly ended in the serial- or no-recall task. Most importantly, we tested whether this time benefit entails the build-up of different types of representations by modeling our data to estimate parameters representing item and binding memory.

**Fig. 7:**
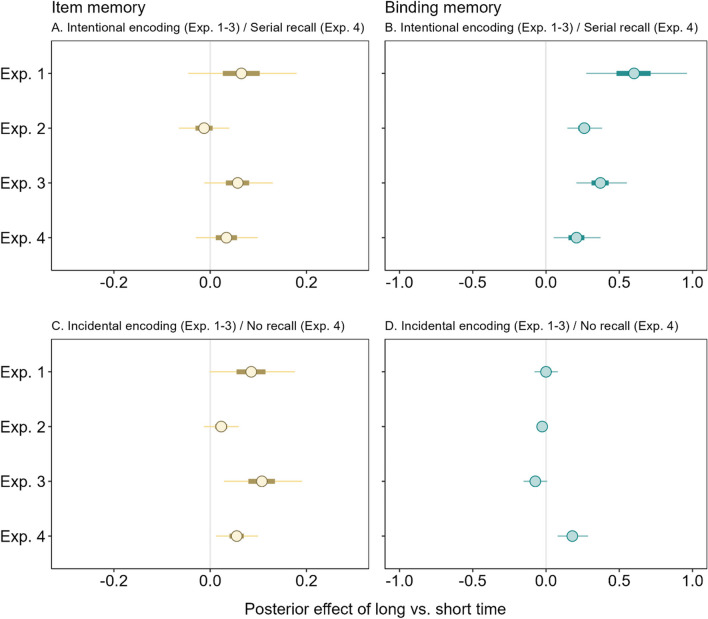
Posterior effect of long versus short free time on item memory (**A** and **C**) and binding memory (**B** and **D**) estimated from the memory measurement model (MMM) fitted to delayed recall across each experiment (Exp.)

As summarized in Fig. 7, our results showed that long uninterrupted time (i.e., during slow span trials in Experiments 1–2 and long free time conditions in Experiments 3–4) improved LTM for both intentional and incidental encoding groups, but for different reasons: Intentional encoding increased binding memory across all the experiments, regardless of the nature of the word-processing task (silent animacy decisions in Experiments 1 and 3–4 or reading aloud in Experiment 2) and whether participants performed an immediate test of WM (Experiment 4). Conversely, there was slight evidence that increased item memory occurred in the incidental encoding group in Experiments 1 and 3 and during no-recall trials in Experiment 4, all of which comprised an elaborative word-processing task (i.e., animacy decisions). As we discuss further on, these results support the notion that time spent actively keeping information in WM is special for episodic LTM because WM is a system that maintains bindings (Oberauer, 2019).

It is also important to note that using cognitive modeling to precisely measure underlying parameters of binding and item memory allowed us to draw these firm conclusions rather than relying only on observed retrieval performance which does not clearly distinguish between different memory processes. For example, in Experiment 3 there was an advantage of long over short free time in both free and serial scoring of delayed performance, but the modeling results showed that this advantage was specific to binding memory. This particular result drives home the point that observed performance (e.g., free scoring) cannot be considered a process pure measure of underlying cognitive processes (e.g., item memory) given that multiple processes can contribute to performance. Using a reconstruction task also included benefits that any differences between the task types were not obfuscated by very low delayed memory performance, and that the nature of the memory test was identical between immediate and delayed testing, following recent prior work (e.g., Loaiza & Lavilla, 2021; Loaiza et al., 2021). Overall, these findings provide novel theoretical insight into why active maintenance in WM is important to the benefits of time for episodic LTM.

### Uninterrupted active maintenance reinforces bindings in working memory

All the experiments showed that intentional encoding of words presented during slow span specifically increased binding memory relative to short maintenance in simple span, and, against our preregistered predictions, also compared with long interrupted maintenance in complex span (Experiments 1 and 2). These results indicate that the long-term benefits of time in WM may be specific to active maintenance during *uninterrupted* free time following studied memoranda. In other words, longer time intervals in WM may specifically help to establish and retain bindings over the long-term when active maintenance can be sustained without distraction, as in slow span, but is less effective to do so when active maintenance is distracted during that the same period of time, as in complex span. These results cohere with views that intentional encoding is important to building contextual associations (Healey, 2018), but also further indicate that active maintenance in WM, in particular, is important if WM serves to establish, update, and dissolve temporary bindings (Oberauer, 2002, 2009, 2019), some of which can be retained in episodic LTM as well. However, the fact that this was specific to slow span conflicts with previous assertions that active maintenance despite distraction during complex span enhances binding memory (e.g., Loaiza et al., 2015), and further suggests that active maintenance in WM may yield different long-term consequences depending on the conditions of the maintenance. We now discuss each of these two major implications in turn.

First, our results speak to a longstanding research question of how active maintenance in WM impacts later retrieval from episodic LTM. Whether participants were required process the words while making silent elaborative animacy decisions (Experiments 1 and 3–4) or simply reading the words aloud (Experiment 2), our results showed that intentional WM encoding during slow span enhanced later binding memory relative to simple span (as well as complex span in Experiments 1–2). This highlights that retention of information from WM to episodic LTM does not only pertain to the individual pieces of information themselves (i.e., item memory), but also to their associated, contextual bindings (i.e., binding memory). Furthermore, the fact that intentional encoding was specific to later binding memory indicates that active maintenance in WM is unique to the benefits of time for episodic LTM because of its role to create these bindings. Thus, the current results are consistent with the binding hypothesis of WM, such that WM is important for other cognitive constructs like LTM due to its capacity-limited role to maintain bindings specifically (Oberauer, 2002, 2009, 2019). For example, recent prior work using the same cognitive modeling approaches used here have shown that varying the number of items to maintain in WM (i.e., set size) impacted binding memory, but not item memory (Bartsch et al., 2019b; Oberauer, 2019). Thus, dissociations of binding and item memory are evident for both set size as well as active maintenance in WM, as we have shown here, pointing to the unique importance of WM for binding memory.

Other recent work has also highlighted the importance of intentional encoding for binding memory in terms of list positions of studied words (Oberauer & Greve, 2022) and item-context associations (Popov & Dames, 2023). For example, Popov and Dames (2023, Experiment 11) showed that the benefit of intentional encoding to retrieval from episodic LTM was specific to remembering word-scene pairings, whereas there was no difference between intentional and incidental encoding groups in the free recall of words. Although these tasks cannot be taken as process-pure measures of binding and item memory, respectively, as we discussed previously, Popov and Dames’ findings cohere with the current results that intentional encoding promotes binding memory, whereas individual items are still available under incidental encoding. In Popov and Dames’ study, however, intentionality was manipulated by instructing participants that there will be a delayed memory test before they were presented words, which does not constrain what participants do in trying to promote episodic LTM. Conversely, here we manipulated intentionality by an immediate memory test and specifically evaluated the role of time in WM to promote episodic LTM by varying the maintenance opportunities across different trial types. This allowed us to elucidate more clearly the role of WM to create durable memory traces. Additionally, manipulating the immediate test within-subjects in Experiment 4 showed the same benefit of time to binding memory regardless of whether participants were tested on the items in WM, indicating that our results are due to the process applied during encoding and maintenance rather than retrieval. Our results therefore fit into an emerging picture that active maintenance over brief periods of uninterrupted time in WM specifically enhances long-term retention of bindings, thereby providing an account of the relationship and transfer between WM and episodic LTM.

The second main implication of our results is that enhanced binding memory particularly occurs due to active and uninterrupted maintenance in WM given that the benefit was specific to slow span and not complex span in Experiments 1 and 2. Thus, in contrast to other work asserting that active maintenance under distraction may increase later binding memory (Loaiza et al., 2015; Loaiza & McCabe, 2012, 2013), we observed only weak evidence that item memory of complex span increased compared with slow span and simple span in Experiment 2. As noted previously, our current experiments closely followed the paradigm of prior work, particularly Experiment 2 of Loaiza and Lavilla (2021), who investigated the use of instructed and spontaneously reported strategies. As we explained previously, when fitting the MMM and MPT models to Loaiza and Lavilla’s data, there was indeed a similar advantage of slow span over simple span and complex span in binding memory, but there was also an advantage of complex span over simple span in binding memory as well (see OSF for details). Thus, it appears that active maintenance in the face of distraction during complex span does not as reliably benefit binding memory as uninterrupted free time in slow span. This may indicate that the distracting processing task disrupts the WM resources required for binding that are available during the uninterrupted free time of slow span. Hence, the relatively greater amount of time compared with simple span may increase the overall availability of the individual items, but the distracting processing task may impair the creation and retention of bindings in WM, consistent with interference-based models of WM and complex span more specifically (Oberauer et al., 2012, 2016). Furthermore, it may be the case that the inconsistent effect of complex span over simple span can be explained by impoverished encoding relative to the simple and slow span conditions (Popov & Reder, 2020). Overall, our results suggest that the long-term retention of information in WM may depend on the nature of active maintenance, such that uninterrupted free time may enhance later binding memory.

A key question remains regarding the specific mechanism driving the long-term benefit of uninterrupted free time to binding memory. As we explained previously, it is unlikely that the benefit is solely attributable to increased time to engage in deep, elaborative strategies given that free time benefits were observed regardless of whether participants were oriented to the semantic characteristics of the words (i.e., the animacy decision in Experiments 1 and 3–4) or simply read the words aloud (Experiment 2). In addition to observing the effect regardless of enforced encoding in the current experiments, Loaiza and Lavilla (2021) also observed a free time benefit regardless of whether reported elaborative spontaneous strategies. Experiment 3 allowed us to address this question regarding the source of the benefit, confirming that varying free time more incrementally yielded corresponding incremental increases to later binding memory under intentional encoding. We take these results to be consistent with two possibilities that are not necessarily mutually exclusive: First, increasing the free time following each word affords more opportunity to recover depleting encoding resources that are important to establishing and retaining bindings in WM, consistent with Popov and Reder’s (2020) resource model. Second, providing free time is likely to be important to stabilizing item–context bindings through short-term consolidation (Cotton & Ricker, 2021). Future research will be required to disambiguate the relative contributions of encoding resources and short-term consolidation to the long-term availability of bindings formed in WM.

Finally, there was some evidence that deep, semantic encoding can enhance item memory even under incidental encoding (Experiments 1 and 3), suggesting that the seminal research in memory that played down the role of intentionality for long-term retention (e.g., Craik & Tulving, 1975; Hyde & Jenkins, 1969, 1973) may be reconciled with more recent work suggesting that intentionality matters (Popov & Dames, 2023). Consistent with Popov and Dames’s (2023) assertion, deeper processing may increase the long-term availability of items, regardless of intentionality, whereas intentional encoding, particularly with uninterrupted free time following, may enhance the long-term availability of bindings. Furthermore, a similar finding of increased item memory with free time during no-recall trials of Experiment 4 suggests that immediate testing of WM may minimize the influence of item memory in the long-term compared with the long-term benefit of free time to binding memory. We anticipate that these will be fruitful avenues of future research.

## Conclusions

How do we retain the information that we currently have active and accessible in mind long after it has left our immediate awareness? Our results suggest that it depends on the nature of what we are doing with that information: Actively maintaining the information in WM (i.e., intentional encoding) may increase the associated, contextual details of the studied information (i.e., binding memory) when there is uninterrupted free time available (i.e., slow span). However, some individual pieces of information may be still remembered in the long-term even if we were not trying to remember it (i.e., incidental encoding), particularly when deep, semantic processes were engaged at the time.

## Data Availability

Please see open practices statement.

## Footnotes

1 Note that, in Experiment 1, many more exclusions were necessary for the incidental encoding group given our preregistered criteria, and thus the remainder of participants collected in this experiment were assigned to the incidental encoding group to achieve the target sample size in each group.

2 Note that an ambiguous word “secretária” (meaning both “secretary” and “desk” in Portuguese) was mistakenly included in the list but was only relevant for an animacy decision on 0.25% of all the trials of the whole experiment, and so it was retained in the analyses.

3 Note that we had written 1.25 s in the preregistration of Experiment 1, but this was an uncorrected typo from an earlier version. Testing the experiment out on ourselves prior to data collection prompted us to extend the presentation duration of the words to make the animacy decision feasible for the participants, but we failed to update the preregistration thereafter.

4 Although participants reconstructed the original order of the words during the immediate and delayed tests, they are not traditional reconstruction-of-order tasks per se. Reconstruction-of-order tasks show all the presented items, without any lures, to be retrieved in their original order, and thus more strongly emphasize memory for order over item memory given that the identity of the items does not need to be retrieved (e.g., Healy, 1974; Neath, 1997; Whiteman et al., 1994). Conversely, the reconstruction tests used here required participants to distinguish the presented words from never-presented lures while recalling the words in their original order, thereby allowing us to distinguish the contributions of item memory and binding memory, as we explain further on.

5 As can be seen in Table 1, a large number of participants in the incidental encoding group were excluded on this basis. We realized that the question was likely confusing because participants could have interpreted it to mean that they tried to retrieve the words during the delayed task (a task that all participants performed, regardless of group assignment) rather than study and encode the words during the immediate task (as we had intended it to mean). As we explain further on, the subsequent experiments addressed this issue by making a clearer delineation between the different “phases” of the experiment for ease of reference during the final survey, and indeed the number of exclusions on this basis was greatly reduced thereafter. An additional exploratory analysis with these excluded participants showing a similar pattern of results as the retained participants in the incidental encoding group in Experiment 1 is reported on the OSF.

6 Note that our preregistrations specify logistic Bayesian mixed effects models as the method of analysis, which we have conducted and can be found on the OSF. However, following reviewer guidance, we present simpler BANOVA results for all the observed performance measures to better focus on the modeling results.

7 The files were categorized accordingly: Speech matched the presented word/arithmetic problem (i.e., correct response; 70% of files), no speech (22% of files), another word said (7% of files), participant said/did something else (0.2% of files), or the speech file was empty (i.e., the experiment failed to record any audio; 1% of files).

8 Given that there are 360 memoranda, we must have one fewer trial per cell of the design compared with the previous experiments (i.e., 4 targets + 4 new WM options + 4 new LTM options for the trials ending in serial recall, and 4 targets + 4 new LTM options for the trials ending in the no-recall parity task × 9 trials each = 360 total options).
